# Oral bacterium contributes to periodontal inflammation by forming advanced glycation end products

**DOI:** 10.1128/iai.00560-24

**Published:** 2025-04-02

**Authors:** Rajendra P. Settem, Ashu Sharma

**Affiliations:** 1 Department of Oral Biology, School of Dental Medicine, University at Buffalo 67227, Buffalo, New York, USA; Georgia Institute of Technology, Atlanta, Georgia, USA

**Keywords:** *Tannerella forsythia* (*T. forsythia*), methylglyoxal (MGO), advanced glycation end products (AGEs), human gingival fibroblast (HGF), alveolar bone loss, endothelium dysfunction

## Abstract

The oral bacterium *Tannerella forsythia* is associated with periodontitis, an inflammatory disease affecting tooth-supporting tissues. The bacterium produces a dicarbonyl compound, methylglyoxal (MGO), whose levels correlate with the severity of periodontitis. MGO can induce inflammation directly or via the generation of glycation products called advanced glycation end products (AGEs). *T. forsythia*-produced MGO has been shown to cause tissue collagen glycation, which in turn can induce pro-inflammatory cytokine secretion in monocytes via receptor for advanced glycation end product (RAGE) receptor activation. The current study investigated the impact of *T. forsythia*-secreted MGO on human gingival fibroblasts and endothelial cells. For assessing the *in vivo* impact of *T. forsythia*-secreted MGO, we employed an oral gavage-induced mouse model of periodontitis utilizing the wild-type and MGO-deficient strains of *T. forsythia*. Our results showed that the apoptotic activity was enhanced, and cell migration was reduced in fibroblasts exposed to collagen treated with the *T. forsythia* wild-type culture supernatant. Moreover, monocyte binding, reactive oxygen species production, and inflammatory cytokine secretion were increased in fibroblasts, and neutrophil transendothelial migration was enhanced in response to the *T. forsythia* wild type-treated collagen. *In vivo*, increased AGE accumulation in gingival tissues with increased alveolar bone loss was observed in wild-type *T. forsythia* as compared to the MGO-deficient strain-infected mice. These data demonstrated that *T. forsythia*-secreted MGO contributes to periodontal tissue destruction by mitigating gingival fibroblast-mediated tissue healing and promoting endothelial cell dysfunction. These findings provide a basis for targeting the *T. forsythia*-associated AGE-RAGE axis in alleviating periodontitis.

## INTRODUCTION

Periodontitis (PD) is a common bacterially induced inflammatory disease often resulting in tooth loss. A bacterial triad, known as the “red complex,” comprising *Tannerella forsythia*, *Porphyromonas gingivalis,* and *Treponema denticola* is implicated in the development of PD (1, 2). While the red-complex bacteria remain clinically important, advanced detection methods based on 16S rRNA gene sequencing have revealed a more complex polymicrobial etiology for periodontitis, implicating additional pathobiont species such as *Filifactor alocis*, *Prevotella intermedia*, and members of the TM7 phylum, known as Saccharibacteria. The findings suggest a new model of pathogenesis where polymicrobial synergy and dysbiosis, rather than specific pathobiont bacteria, underlie the pathogenesis of periodontitis (3). Previous studies have shown that the severity of PD, characterized by an increase in periodontal pocket depth and loss of alveolar bone, correlates with the subgingival load of red-complex bacteria. Additionally, the levels of methylglyoxal (MGO) in diseased pockets increase with the disease’s severity and follow the subgingival load of red-complex bacteria (2). *T. forsythia* has the unique ability to produce MGO, an electrophilic and highly reactive dicarbonyl compound which can covalently modify amino acid side chains in proteins to generate inflammatory adducts known as advanced glycation end products (AGEs) (4), which can promote inflammation via receptor for advanced glycation end product (RAGE) activation. Additionally, MGO can directly modify and disrupt the integrity of host defense mediators such as anti-microbial peptides. MGO can also be toxic to bacteria and covalently modify the structural and biological activity of virulence factors and adhesins. Thus, *T. forsythia*-produced MGO might contribute to the inflammatory processes and dysbiosis associated with periodontitis.

*T. forsythia* is likely one of the few bacteria among the subgingival community implicated in periodontitis which has the ability to synthesize MGO via an enzymatic pathway dependent on methylglyoxal synthase (MgsA), which converts the glycolytic intermediate dihydroxyacetone phosphate into methylglyoxal (4, 5). A BLASTp search of the Human Oral Microbiome Database indicated that the oral bacterium *Treponema socranskii* possesses a homolog of the MgsA enzyme as well. The *T. socranskii* MgsA homolog shares a 73.3% amino acid identity with the Tf MGO (97% coverage spanning 153 amino acids). MGO accumulation is associated with several chronic inflammatory diseases such as atherosclerosis, diabetes, and aging-related diseases (6–9). In these diseases, MGO accumulates in the body mainly as a by-product of the host glycolysis and/or secretion by the gut microbiota (10–12). AGEs are involved in the inflammatory process by activating RAGEs on macrophages, gingival fibroblasts (GFs), and endothelial cells, thereby inducing the release of inflammatory cytokines.

The role of MGO secreted by *T. forsythia* in periodontal inflammation has not been explored. In PD, as mentioned above, the disease severity has been shown to correlate with levels of MGO and red-complex bacteria, including *T. forsythia* (2). However, the role of microbially secreted methylglyoxal on periodontal tissues has yet to be explored. Human gingival fibroblasts (HGFs) and periodontal ligament fibroblasts are crucial for maintaining gingival tissue integrity and immune surveillance against external stimuli, including bacterially derived factors. The cytokine responses and cellular behavior of HGF cells are altered when exposed to MGO or MGO-modified matrix proteins such as collagen. MGO-modified collagen has been shown to enhance gingival fibroblast apoptosis and TIMP-1 expression (13). Our previous study showed that *T. forsythia* culture exposed collagen-induced inflammatory cytokine secretion in human monocytic cells (THP-1) via RAGE activation (5).

To gain a better insight into the role of *T. forsythia*-secreted methylglyoxal on periodontal inflammation, we investigated the impact of *T. forsythia*-secreted methylglyoxal on HGFs and endothelial cells. We found that *T. forsythia*-secreted methylglyoxal and AGE adducts present in the culture supernatant of *T. forsythia* induced cell apoptosis and reactive oxygen species (ROS) production in HGFs. In addition, *T. forsythia*-modified AGE collagen caused higher expression of ICAM1 and VCAM adhesion molecules in HGFs, which led to increased binding of monocytic cells to the HGFs. Moreover, we observed that in the presence of *T. forsythia*-modified AGE collagen, HGF migration was significantly compromised, and neutrophil transmigration across endothelial monolayer was enhanced. In a mouse model of oral gavage-induced periodontitis, more AGE accumulation and alveolar bone loss were observed when mice were infected with the wild-type *T. forsythia* compared to the MGO-deficient mutant. Overall, our results indicate that *T. forsythia*-secreted MGO contributes to periodontal tissue damage by promoting inflammation, mitigating tissue healing, and promoting endothelial dysfunction.

## MATERIALS AND METHODS

### Bacterial growth and culture conditions

*T. forsythia* was grown in TF broth (brain heart infusion media containing 5 µg/mL hemin, 0.5 µg/mL menadione, 0.001% *N*-acetylmuramic acid, 0.1% L-cysteine, and 5% fetal bovine serum) as liquid cultures or on plates containing 1.5% agar in broth under anaerobic conditions as described previously (14). An isogenic *T. forsythia* mutant, TFM-1726, with the *mgsA* gene encoding methylglyoxal synthase inactivated, was generated and grown in broth or agar plates containing 5 µg/mL erythromycin, as we have described previously (5). TFM-1726 gene deletion mutant was generated by insertional inactivation of MgsA homolog BFO_1726 in the *T. forsythia* ATCC-43037 with an erythromycin cassette (15, 16). DNA sequencing of the insertion site flanking region and quantitative reverse transcription-PCR were performed to confirm correct integration and to rule out polar effects.

### Flow cytometry

Apoptosis in HGFs was monitored by flow cytometry with an Annexin V detection kit according to the manufacturer’s protocol (BD Biosciences Pharmingen, San Diego, CA, USA). Briefly, HGFs were seeded into a 12-well plate (0.5 × 10^6^ cells/well) and incubated overnight. After 12 h, cells were incubated with 25 µL or 50 µL of cell-free culture supernatants from either the Tf-43037 or TFM-1726 strain. After 2 h, the cells were washed with cold phosphate-buffered saline (PBS) and detached with 0.05% trypsin in EDTA, centrifuged at 1,000 × *g* for 5 min, and washed once with PBS. The cells were then resuspended in a binding buffer with fluorescein isothiocyanate (FITC)-labeled Annexin V and kept on ice for 15 min. Propidium iodide (PI) was added, and the cells were incubated for another 30 min on ice. Finally, the samples were analyzed using flow cytometry (FACS Calibers; Becton-Dickinson, San Jose, CA, USA).

### THP1 immune cell binding to HGF monolayer

Human monocytic cells (THP-1) were cultured in RPMI-1640 complete medium. The cell number was adjusted as 1 × 10^6^/mL and labeled with fluorescein-5-succinimidyl ester (Thermo Fisher). 24-well cell culture plate was coated with Tf-43037- or TFM-1726-treated collagen-1 (10 and 20 µg/well) as we described previously (5). TFB medium alone and commercial MGO-modified collagen were used as negative and positive controls, respectively. Human gingival fibroblast cells were monolayered on collagen-coated wells overnight. Then FITC-labeled THP-1 cells were added to the wells and incubated for 30 min. The wells were washed with PBS three times to wash unbound THP-1 cells, and the attached FITC-labeled THP-1 cells were observed with an inverted fluorescence microscope. The FTIC intensity was measured by Flex Station 3 Multi-Mode Microplate Reader (Molecular Devices)

### Quantitative real-time polymerase chain reaction

A collagen-coated 48-well cell culture plate was incubated with Tf-43037 or MgsA isogenic mutant TFM-1726 culture supernatant for 24 h to allow formation of AGEs (AGE-collagen). After washing the wells with PBS, human gingival fibroblasts were seeded at 0.5 × 10^5^ cells per well. After 6 h, HGFs were detached, and total RNA was extracted using the RNeasy Mini Kit per manufacturer instructions (Qiagen). cDNA synthesis was performed with total RNA using the Superscript cDNA Synthesis Kit (TaKaRa Bio) according to the manufacturer’s instructions. cDNA was then used to estimate the relative levels of adhesion molecule transcripts with a SYBR green PCR mix (Bio-Rad). ICAM1, VCAM, and glyceraldehyde-3-phosphate dehydrogenase (GAPDH) primer sequences are listed in Table 1. PCR conditions were as follows: 1 cycle at 95°C for 30 s followed by 40 cycles at 95°C for 5 s and at 60°C for 30 s, using the CFX96 Real-Time PCR Detection System (Bio-Rad). The relative mRNA levels of ICAM1 and VCAM were analyzed by 2^–ΔΔCt^, and results were expressed in fold change relative to HGFs with medium alone. The gene expression was normalized with GAPDH, used as an internal control. No template blank served as the negative control.

**TABLE 1:** List of primers and their sequences

Gene	Forward	Reverse
ICAM1	5′-AGCGGCTGACGTGTGCAGTAAT-3′	5′-TCTGAGACCTCTGGCTTCGTCA-3′
VCAM	5′- GATTCTGTGCCCACAGTAAGGC-3′	5′- TGGTCACAGAGCCACCTTCTTG-3′
GAPDH	5′- CCTGCACCACCAACTGCTTA-3′	5′- GGCCATCCACAGTCTTCTGAG-3′

### Assessment of cytokines and protein glycation adducts in culture supernatants

HGFs were incubated with collagen modified with either culture supernatant from Tf-43037, TFM-1726, or TFB medium alone for 16 h. According to the manufacturer’s instructions, culture supernatants were collected, and ELISAs were performed using the Bio-Plex-MAGPIX Pro and ELISA kits (Life Technologies Corporation, Frederick, MD, USA). Levels of AGEs in bacterial culture supernatants were determined by using the OxiSelect Advanced Glycation End Product Competitive ELISA Kit (catalog number STA-817, Cell Biolabs, Inc., San Diego, CA, USA) as per manufacturer instructions. The concentrations of AGEs were calculated using the standard curve of AGE-bovine serum albumin (BSA). All data are shown as mean ± standard deviation from two to three independent experiments with two technical replicates each time.

In addition, we performed SDS-PAGE followed by silver staining to evaluate the protein profiles of culture supernatants of Tf-43037 and TFM-1726 strains. For this purpose, Tf wild type and MGO mutant TFM-1726 were grown as described previously (17). Ten milliliters of each bacterial culture from the mid-log phase was centrifuged at 4,500 × *g* for 15 min. Supernatants were collected, and proteins were precipitated overnight at 4°C in 80% ammonium sulfate saturation. The samples were then centrifuged at 10,000 × *g* for 30 min at 4°C, and precipitates resuspended in cold PBS were dialyzed extensively against PBS at 4°C. The protein samples were visualized after separation on a 10% SDS-PAGE gel before silver staining, as described previously (18).

### Luminol ROS detection

The luminol-based chemiluminescence assay for detecting ROS was used as previously described (19). Briefly, HGF cells were incubated with 250 µg/mL horseradish peroxidase (HRP) (Alfa Aesar) for 15 min to allow for fluid-phase uptake into the endosomal system prior to incubation with *T. forsythia*-modified collagen-1 coated wells. The cells were placed in Hank’s balanced salt solution (HBSS) (Corning) with 250 µg/mL HRP and 50 µM luminol. Luminescence was read on a Synergy temperature-controlled plate reader (Bio-Tek) by reading once per minute, with a 1,000 ms integration time, for 90 min at 37°C.

### Scratch wound healing assay

The effect of Tf-secreted MGO on cell migration was determined by scratch wound assay as described previously (20). Briefly, HGFs were seeded at 0.5 × 10^5^ per well in a 24-well plate. After reaching 60% cell confluence, the cell monolayer was scratched with a 10 µL pipette tip. Following the scratch, the wells were washed with PBS to remove detached cells and cell debris. The wells were then treated with 50 µL of cell-free bacterial culture supernatants from Tf-43037 and TFM-1726. After 24 h, digital images were captured using a camera-equipped inverted microscope (Carl Zeiss, Inc., Thornwood, NY, USA) using LAS-X Leica image acquisition software (Leica Microsystems, Wetzlar, Germany). Wound width was determined by measuring the distance between scratch edges by ImageJ software (Software 1.48q; Rayne Rasband, National Institutes of Health, USA). Net wound closure was calculated by subtracting the wound width at 24 h from the width at 0 h. The percentage of closed wound area was calculated using the following formula:


$$\frac{Wound\;closure\;area\;at\;0\;h-\;wound\;closure\;area\;at\;24\;h}{wound\;closure\;area\;at\;0\;h}\times 100.$$

Assays were performed three times on independent days using triplicate wells.

### Immunofluorescence staining

Accumulation of AGEs was determined by immunofluorescence staining. Mouse maxillary jawbone slides were de-waxed, rehydrated, and rinsed with PBS three times for each 10 min. Microwave-based antigen retrieval was performed in 10 mM citrate buffer (pH 6.0). These slides were incubated with 1% BSA (PBS) for 30 min. Sections were incubated with anti-AGE (1:50, ab23722; Abcam, Cambridge, MA, USA) rabbit polyclonal antibodies at 4°C overnight. The binding of the primary antibody was localized with the Alexa Fluor 488-conjugated anti-rabbit secondary antibody (Jackson ImmunoResearch Laboratories, West Grove, PA, USA) for 30 min. The negative control consisted of Alexa Fluor 488-conjugated mouse IgG incubated without primary antibody treatment. Slides were mounted with DAPI mounting medium and examined using a Zeiss LSM510 (Carl Zeiss, Inc.) confocal microscope at the University at Buffalo core facility (optical imaging analysis facility).

### Micro-computed tomography

Alveolar bone loss was determined in the *T. forsythia*-induced periodontitis mouse model by oral gavage, as described in our previous publication (14). Briefly, mice divided into three groups (eight mice per group) received kanamycin (1 mg/mL) in drinking water for 4 days followed by 3 days of antibiotic-free water. In group 1 (sham group), mice were given only vehicle (2% carboxymethyl cellulose [CMC]) for 2 weeks at 48 h intervals. Groups 2 and 3 were infected by oral gavage with a 100 µL suspension in 2% CMC of 1 × 10^9^ CFU/mL of Tf-43037 and MGO-deficient mutant strain, TFM-1726, respectively, every 48 h for 2 weeks. After 6 weeks of the first infection, the mice were euthanized, and the entire mouse head was harvested and fixed in 4% formaldehyde for high-energy micro-CT (Scanco100 μCT), and scans were obtained as described previously (21, 22). Images were reconstructed and analyzed using Analyze Pro software (Analyze Direct, Inc., Kansas, USA) to calculate the distance (micrometers) between the CEJ and ABC, as described previously (14, 17). The net bone loss was calculated by subtracting the sham (group G1) values from those of the groups infected with Tf-43037 or TFM-1726.

### Neutrophil transmigration assay

Human aortic endothelial cells (HAoECs) (5 × 10^4^) were seeded for 48 h in the upper chamber of a transwell culture dish with a 3.0 µm pore size membrane. The endothelial monolayer formed in the upper chamber was treated with 50 µL of either the cell-free culture supernatant from the wild-type Tf-43037 strain or TFM-1726 strain for 3 h before adding neutrophils. Human neutrophils were isolated from peripheral healthy human blood samples using 1-Step Polymorphs solution (Accurate Chemical & Scientific, Carle Place, NY, USA), and they were confirmed by Alexa Fluor 647 mouse anti-human CD66b (BD Biosciences Pharmingen) using flow cytometry as previously described (23). After the treatment, the culture medium was removed, and 100 µL of 1 × 10^6^/mL carboxyfluorescein succinimidyl ester (CFSE)-labeled neutrophils in RPMI were added to the upper chamber, and 1 µM N-formyl-methionine-leucyl-phenylalanine (fMLP) in RPMI was added to the bottom neutrophil-receiving chamber. Neutrophils from the bottom chamber were collected and centrifuged at 300 × *g* for 10 min at 4°C. Neutrophils were resuspended in 50 µL of HBSS, placed on coverslips for 15 min at room temperature in the dark, and then fixed by adding 50 µL of 4% paraformaldehyde (PFA) for 10 min. Coverslips were directly mounted on glass slides with a fluorescence mounting medium, imaged using fluorescence confocal microscopy, and quantified at five random fields. Commercial MGO (10 µM) and TF broth (25 µL) were used as positive and negative controls, respectively.

### Statistical analysis

All data are shown as mean ± standard deviation from two to three independent experiments with three replicates. Group comparisons were made using analysis of variance, followed by paired or unpaired *t*-tests as appropriate (indicated in the figure legends) using GraphPad Prism version 9 (GraphPad Inc.). For all tests, the statistical significance was defined as *P* < 0.05.

## RESULTS

### *T. forsythia*-secreted methylglyoxal induces cell necrosis and apoptosis

**Fig 1:**
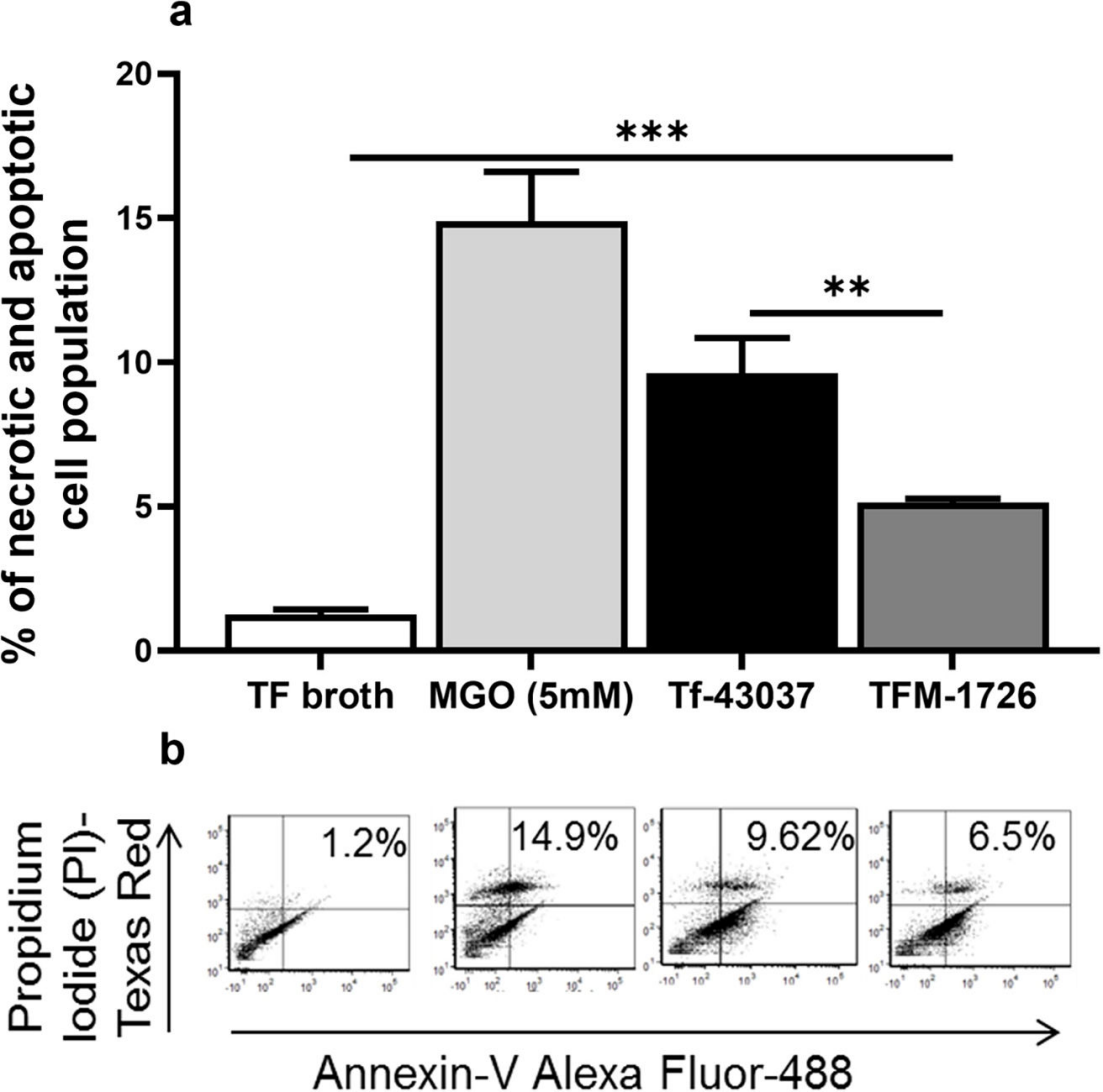
Effect of *T. forsythia-*secreted MGO on gingival fibroblast apoptosis and necrosis. HGFs were seeded into a 12-well cell culture plate (0.5 × 10^6^ cells/well) and incubated overnight. Cells were treated for 2 h with 25 µL and 50 µL of cell-free culture supernatants from Tf-43037 or TFM-1726, TFB (20% vol/vol), and MGO (5 mM). After washing the wells with cold PBS, cells were detached with trypsin-EDTA solution, collected by centrifugation, and stained with FITC-labeled Annexin V and propidium iodide (PI). Results are representative of three independent experiments. (a) The bar graph shows the percentage of positive cell population for Annexin V and PI. (b) Scatter plots show the representative flow cytometry images from each experimental condition. Bars represent mean ± SD. ***P < 0.001, **P < 0.01.

**Fig 2:**
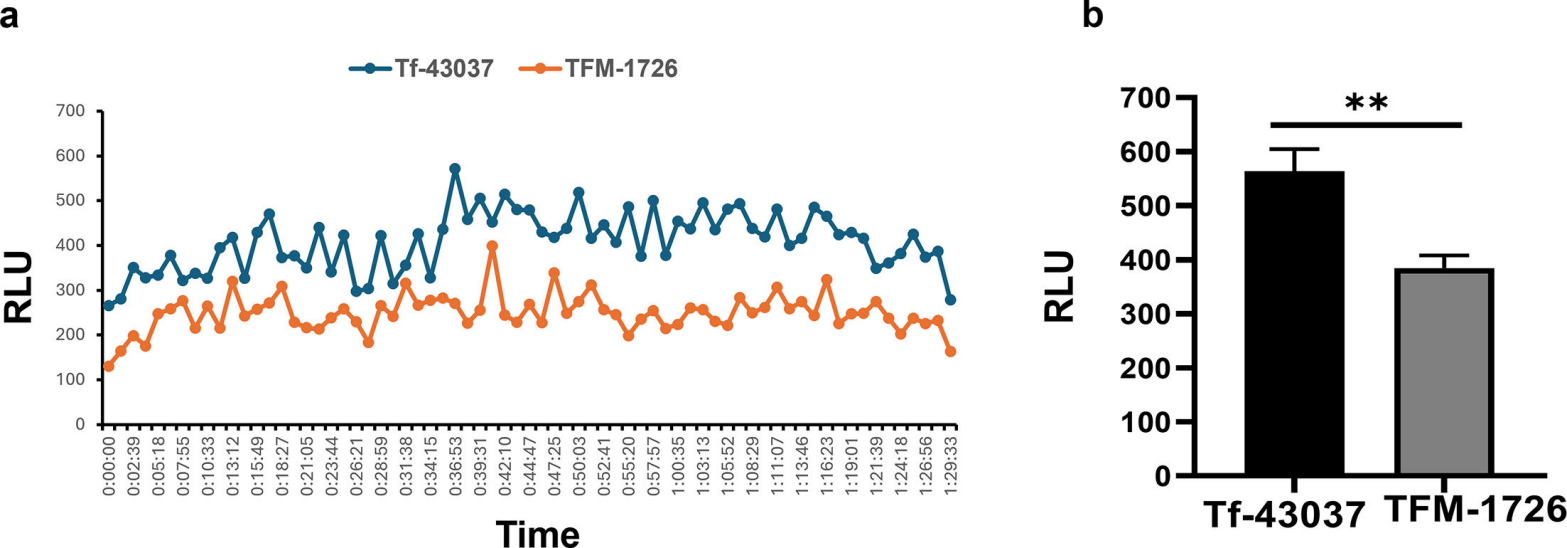
*T. forsythia*-secreted MGO causes ROS production in human gingival fibroblasts (HGFs). Luminol-based ROS detection was used to quantify the ROS production by HGFs in response to the cellfree culture supernatants from Tf-43037 or TFM-1726. (a) Representative panel for time course of luminol luminescence detection, shown in relative luminescence units (RLU). (b) Mean peak RLU (with standard deviation) detected over three independent experiments. **P <0.01 (paired *t*-test).

The impact of *T. forsythia*-secreted methylglyoxal on HGFs was assessed by assaying for apoptosis and necrosis in HGFs exposed to culture supernatants of *T. forsythia* wild-type and MGO-deficient mutant strains. Our previous study showed that cell-free culture supernatant from *T. forsythia* can generate collagen glycation adducts (AGE-collagen) that can induce the secretion of inflammatory cytokines by monocytes via RAGE activation. Moreover, we show that proteins in the culture supernatants are modified by MGO secreted by *T. forsythia*. AGE adducts were identified in significantly higher amounts in the culture supernatant of the wild-type strain than in the MGO-deficient mutant (TFM-1726) strain (Fig. S1). The impact of AGE adducts formed in the culture supernatant on HGFs was determined. Our data showed that as compared to the TFM-1726 derived culture supernatant, *T. forsythia* wild-type culture supernatant induced significantly elevated apoptosis and necrosis in HGFs as judged by Annexin V staining and propidium iodide uptake (Fig. 1a and b). To determine the likely cause of apoptosis in HGFs, we analyzed the ROS production in HGFs. MGO and glycated adducts formed by MGO are known to induce ROS production in cells through RAGE-dependent induction of multiple pathways, including the activation of NADPH oxidase (24–27). Since ROS is often linked to apoptosis in cells (PMID: 11256882), we compared the production of ROS in HGFs in response to the wild-type Tf-43037 and TFM-1726 culture supernatants by a luminol-based ROS detection assay. We observed that as compared to the culture supernatant from the wild-type strain, the culture supernatant from the TFM-1726 strain induced significantly lower ROS production during the time course, and the peak ROS production aligned with gingival fibroblast apoptosis and necrosis (Fig. 2a and b). In support of the role of ROS in HGF apoptosis, LPS administration in a mouse model has been shown to cause gingival fibroblast apoptosis via oxidative stress (28). Taken together, we suggest that Tf -MGO-induced ROS production is likely responsible for the apoptosis in HGFs.

### *T. forsythia* methylglyoxal production promotes monocyte adhesion to fibroblasts

**Fig 3:**
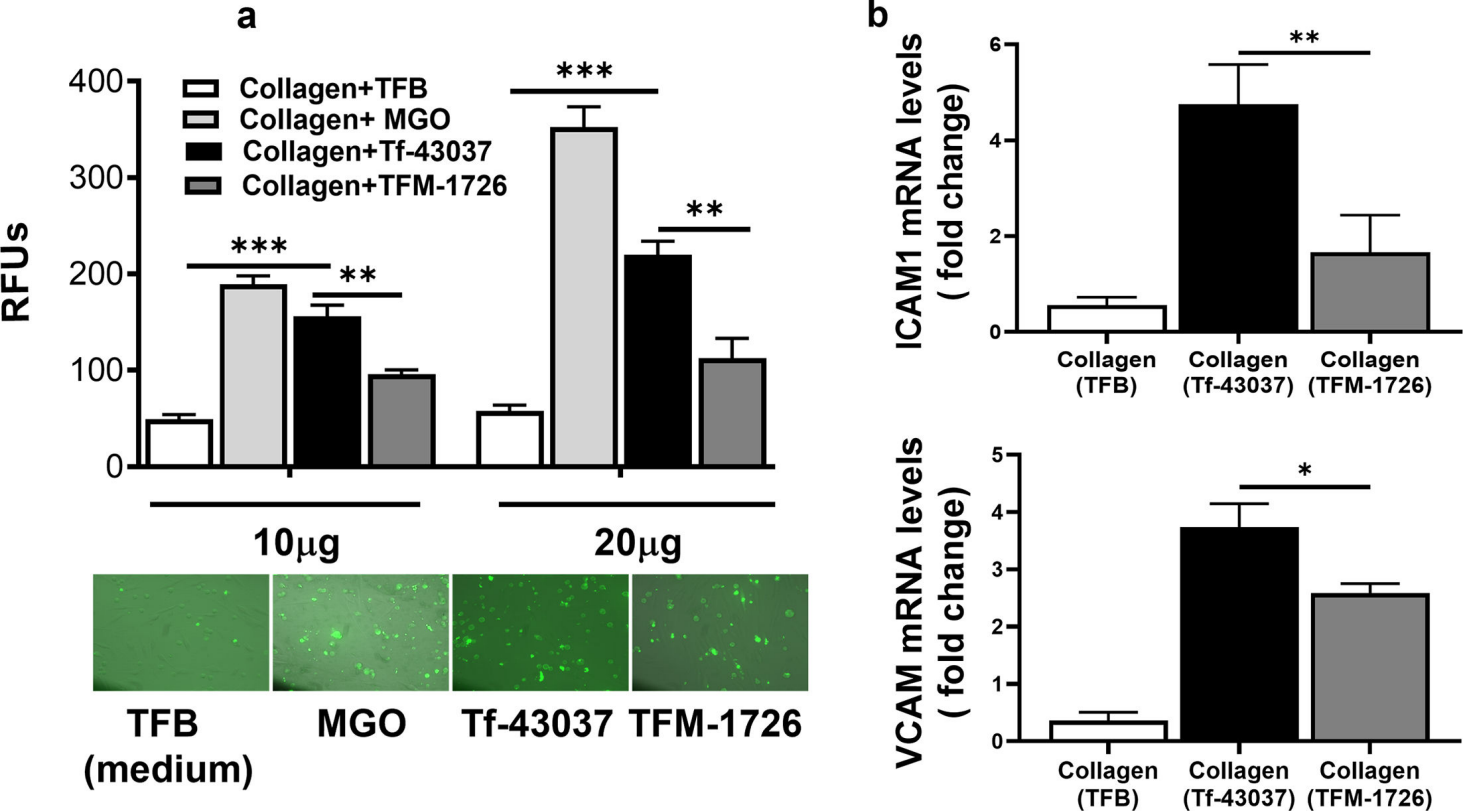
*T. forsythia*-MGO-modified collagen-AGE accelerates the binding of monocytes to HGFs. Collagencoated wells (10 and 20 µg per well) were treated with cell culture supernatants. HGF monolayers were then formed on collagen-coated wells, and fluorescein-5-EX succinimidyl ester-labeled THP-1 cells were incubated for 2 h on cell monolayers. Unbound cells were washed with PBS, and monocyte binding was visualized by fluorescence microscopy. Relative expression levels of ICAM1 and VCAM transcripts were quantified by quantitative reverse transcription-PCR. (a) Fluorescence emission; bars represent mean ± SD (top panel) and representative images (lower panel). (b) Fold change in gene expression of ICAM1 and VCAM relative to HGFs with medium alone. Bars represent mean ± SD. *P < 005, **P < 0.01, ***P < 0.001.

**Fig 4:**
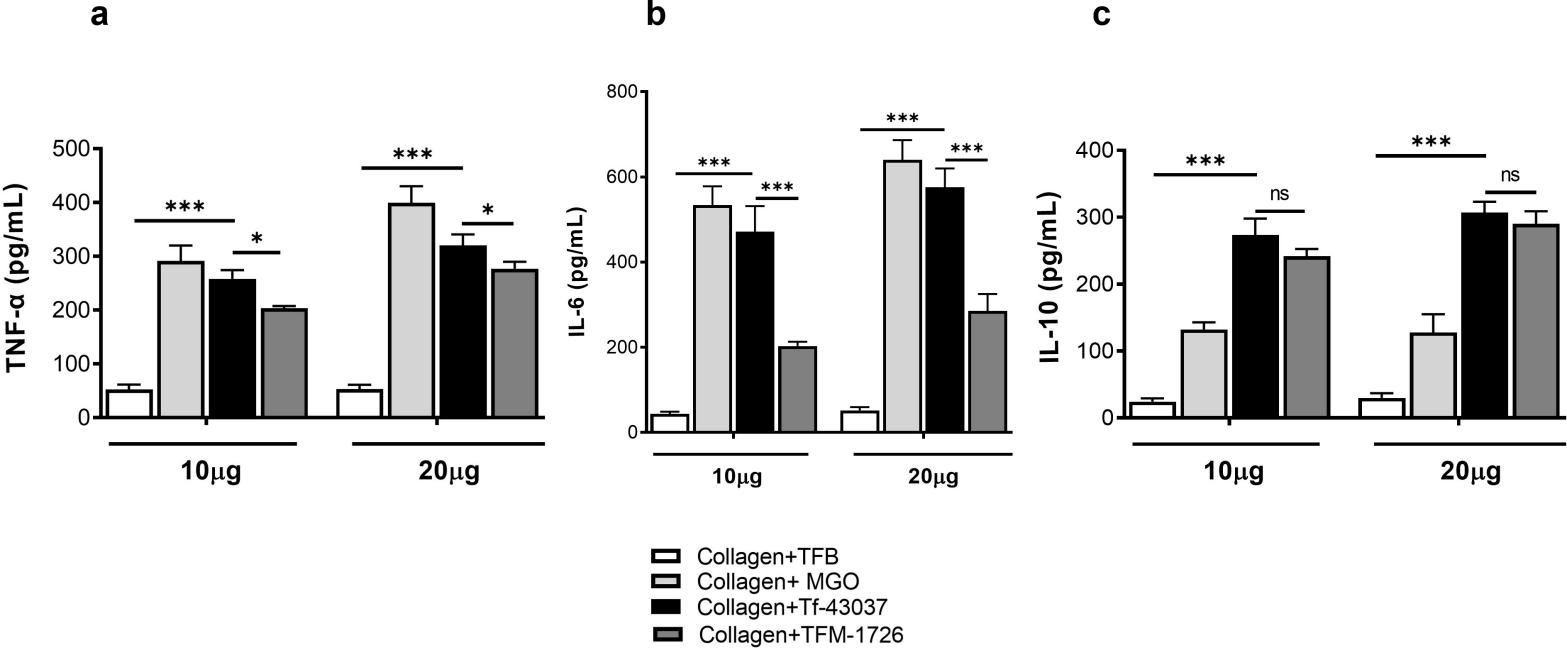
*T. forsythia*-secreted MGO via formation of collagen AGEs contributes to cytokine production by HGFs. *T. forsythia*-MGO-modified collagen-AGE induces higher amounts of (a)TNF-α and (b) IL-6 than mutant strain TFM-1726, but the difference was not significant in (c) IL-10 levels. Cytokine levels were quantified with Bio Plex-MAGPIX (Bio-Rad Inc., USA). ***P < 0.001 vs TFB‐treated collagens. *P < 0.05, ***P < 0.01 vs TFM-1726-treated collagen. ns, not significant.

Immune cell infiltration and accumulation in the gingival tissue are essential in the progression of periodontal disease. MGO-derived AGEs can increase the severity of inflamed periodontal sites by increasing the adhesive activity of immune cells to gingival fibroblasts. It has been shown that MGO-exposed bovine serum albumin promotes immune cell binding to the gingival cells, suppressing immune cell trafficking and surveillance (29). We compared the effect of the *T. forsythia* wild-type and TFM-1726 culture supernatant-treated collagen on HGFs’ ability to recruit and concentrate THP-1 monocytes. Collagen is a rich component of gingival tissue and plays a significant role in tissue remodeling. The data showed that the ability of THP-1 monocytes to adhere to HGFs was significantly enhanced when HGFs were plated on collagen pre-treated with the wild-type culture supernatant as compared to the TFM-1726 culture supernatant. (Fig. 3a). These changes correlated with the expression levels of major cell adhesive molecules ICAM1 and VCAM in HGFs (Fig. 3b). MGO and MGO-modified protein adducts have been shown to increase the expression of these adhesive molecules via RAGE activation (30). In addition, the major pro-inflammatory and pro-osteoclastogenic cytokine tumor necrosis factor-alpha (TNF-α) and interleukin (IL)-6 levels were significantly induced in HGFs in the presence of Tf-43037 modified collagen over TFM-1726 modified collagen (Fig. 4a and b). We detected lower levels of IL-10 with no significant difference between the two conditions (Tf-43037 vs TFM 1726) (Fig. 4c). However, the significant amount of IL-10 production from HGFs in the presence of MGO over TFB alone could be a part of the defensive mechanism to counteract the pro-inflammatory effect of MGO or AGE derivatives (31). Together, these data show that *T. forsythia*-secreted MGO can trigger inflammation in gingival tissues via accumulation of monocytes and activation of inflammatory cytokines by fibroblasts as well as monocytes, as we have shown previously (5).

### *T. forsythia* methylglyoxal secretion mitigates gingival fibroblast migration

**Fig 5:**
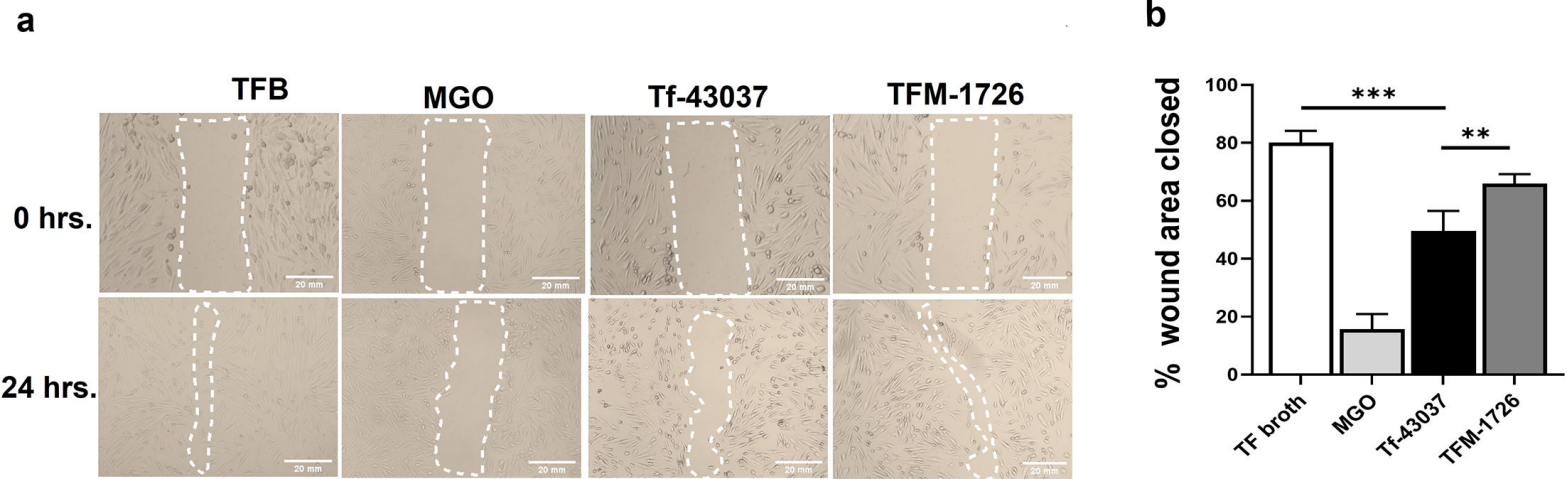
*T. forsythia-*formed AGEs impair gingival fibroblast cell migration. HGF monolayers in culture wells were scratched with a pipette tip to create a wound. (a) Wells were then incubated with the cell-free bacterial culture supernatant from Tf-43037 or TFM-1726 for 24 h. The wound width from collected images was determined by measuring the distance between the edges of the scratch with ImageJ software. (b) Data are expressed as the mean values of percentage wound closure relative to 0 h of respective treatment and represent the mean percentage closure ± SD (*n* = 3). **P < 0.01, ***P < 0.001. MGO and TFB were used as positive and negative controls, respectively. Assays were performed three times on independent days using triplicate wells.

Previous studies have shown that glycation of extracellular matrix proteins, mainly collagen, by MGO impacts fibroblasts’ adhesion and proliferation ability, eventually compromising wound healing (32, 33). In this study, we utilized a cell scratch assay to assess the effect of *T. forsythia*-MGO-modified collagen on HGF migration. Our data showed that HGFs seeded on collagen treated with the wild-type strain supernatant were unable to migrate and close the scratch (wound) less effectively than the fibroblasts plated on collagen treated with either the broth alone (TFB) or the TFM-1726 culture supernatant (Fig. 5a and b). These observations suggest that *T. forsythia* can compromise gingival tissue integrity via the accumulation of MGO-modified matrix proteins at periodontitis sites.

### *T. forsythia*-secreted MGO promotes neutrophil transendothelial migration

**Fig 6:**
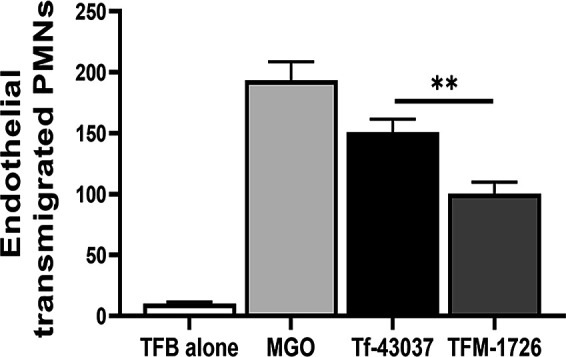
*T. forsythia*-secreted MGO promotes neutrophil trans-endothelial migration. The effect of *T. forsythia* on endothelial function was measured by PMN transmigration assay. HAoECs seeded on transwell membranes (3.0 µm pore size) were treated with cell-free bacterial culture supernatants (Tf-43037 or TFM-1726). CFSE-labeled neutrophils were then added and allowed to migrate for 3 h to the receiving lower chamber containing 1 µM fMLP peptide in RPMI. Migrated neutrophils were resuspended in 50 µL of HBSS, attached to coverslips, and fixed with 4% PFA. Coverslips were mounted on glass slides with mounting medium and imaged using fluorescence confocal microscopy and quantified in five random fields. Commercial MGO (10 µM) and TF broth (25 µL) were used as positive and negative controls, respectively. Data are presented as mean ± SD. **P < 0.01.

We observed increased expression of ICAM-1 (CD54) and VCAM (CD106) adhesion molecules in HAoECs in response to cell-free supernatant from the wild-type strain as compared to that from the MGO-deficient strain (Fig. S2a and b). Next, we were interested in determining if adhesion molecule upregulation in endothelial cells impacted neutrophil transmigration. Circulating neutrophils are captured onto the endothelial cells by upregulated adhesion molecules in response to inflammatory signals due to pathogen-derived factors (34, 35). For this purpose, we performed neutrophil transmigration assays in transwell dishes. We estimated the transmigration of CFSE-labeled polymorphonuclear leukocytes (PMNs) layered on endothelial cell monolayers in top wells with culture supernatants toward fMLP into the bottom wells. We found a significantly higher number of PMNs in the wells in which the endothelial monolayer was treated with Tf-43037 cell-free medium as compared to the TFM-1726 and TFB medium (Fig. 6)

### *T. forsythia* causes AGE accumulation in mouse gingival tissue

**Fig 7:**
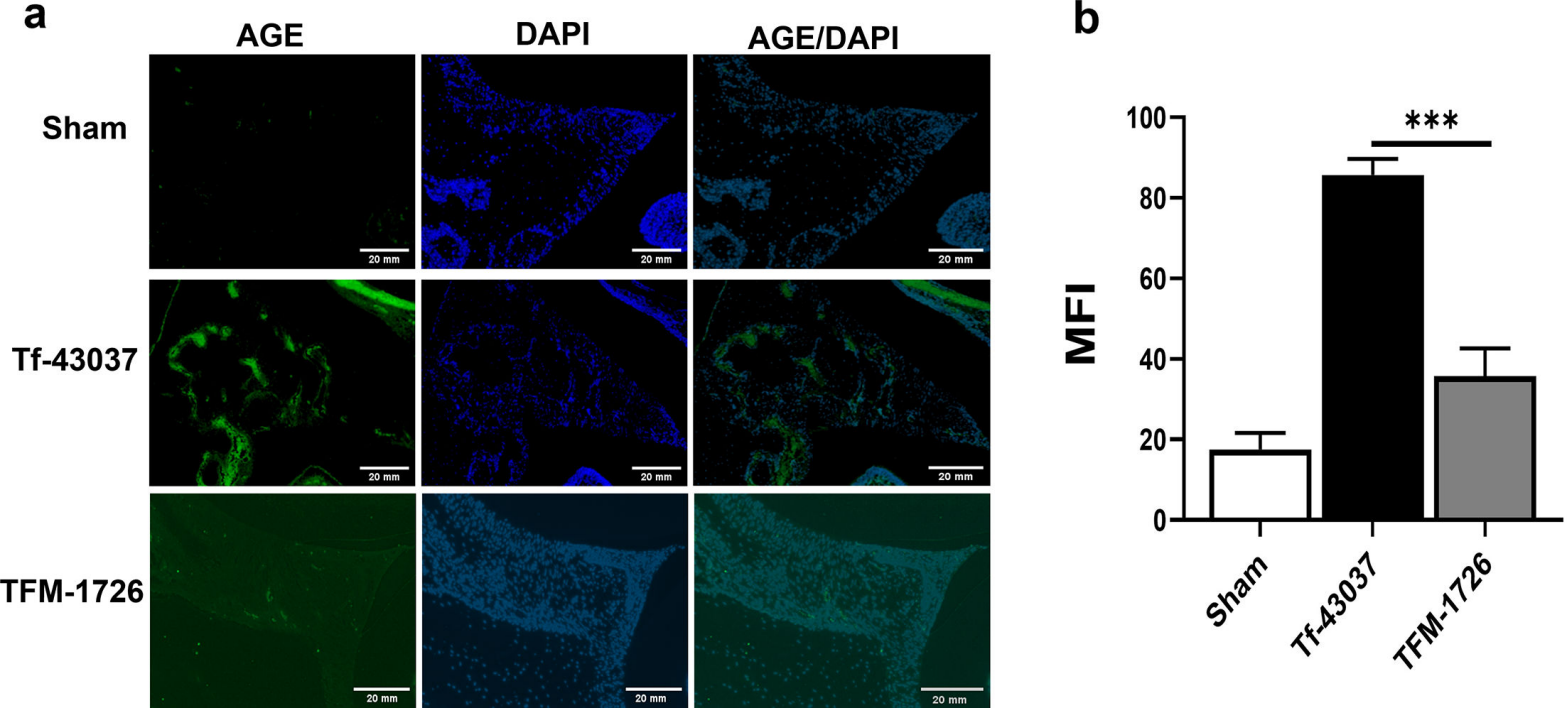
*T. forsythia* causes advanced glycation end product (AGE) accumulation in mouse gingival tissue. (a) *T. forsythia*-induced AGEs in mouse gingival tissues were determined by staining with AGE-specific antibody and Alexa fluor 488-conjugated secondary antibody as described in the main text. Negative control consisted of Alexa Fluor 488-conjugated antibody without primary antibody incubation. Slides were mounted with 4′,6-diamidino-2-phenylindole (DAPI) mounting medium and examined using a Zeiss LSM510 (Carl Zeiss, Inc.) confocal microscope at a University at Buffalo core facility. (b) AGE-positive cells were quantified by using ImageJ and expressed as mean fluorescence intensity (MFI). Representative images are from three independent experiments. ***P < 0.001.

To determine the contribution of methylglyoxal in periodontitis, we tested the virulence of *T. forsythia* wild-type and MGO-deficient strains in an oral gavage-induced alveolar bone loss mouse model of periodontitis as described previously (17). First, to ascertain the role of *T. forsythia* infection in AGE production in gingival tissues, we examined AGE production in mouse gingival tissue following oral infection with the wild-type Tf-43037 or the TFM-1726 mutant strain. Following infection, gingival tissue sections stained with anti-AGE specific antibody were analyzed by immunofluorescence microscopy. The data showed significantly higher AGE accumulation in the wild-type *T. forsythia*-infected group than in the TFM-1726 and sham-infected groups (Fig. 7a and b). Some background immunofluorescence staining observed in the TFM-1726 and sham-infected mice was likely the result of endogenous AGEs derived from non-enzymatic and metabolic pathways of the host, as is well documented (36) (Fig. 7a and b).

### Loss of methylglyoxal production in *T. forsythia* attenuates bacterially induced alveolar bone resorption

**Fig 8:**
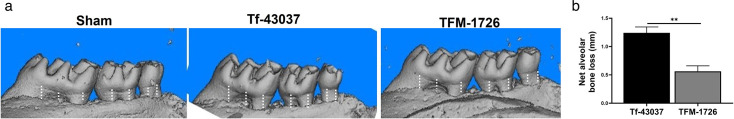
Loss of MGO secretion attenuates *T. forsythia*-induced alveolar bone resorption in mice. Mice (*n* = 8) were infected by oral gavage every 48 h for 2 weeks with 10^9^ cells per dose of either *T. forsythia* wild-type Tf-43037 or MGO mutant TFM-1726 strain. Sham-infected mice received only vehicle (2% CMC suspension). (a) Alveolar bone levels were assessed after 6 weeks of first infection by measuring the distance from the alveolar bone crest (ABC) to the cementoenamel junction (CEJ) at 14 maxillary buccal sites per mouse (R1–R7 = right jaw; L1–L7 = left jaw). (b) Net bone loss induced by WT or MGO mutant was calculated as mean total ABC–CEJ distance of the bacterially infected group minus the mean total ABC–CEJ distance of the sham-infected group. The data show that the net bone loss caused by the WT strain is significantly higher than that by the MGO mutant. Bars indicate means and standard deviations. Data were analyzed by a Mann–Whitney unpaired *t*-test, and statistically significant differences are indicated as **P <0.01.

The micro-computed tomography analysis of mouse jaw bones showed that the group of mice infected with the MGO-deficient strain exhibited significantly less alveolar bone loss as compared to that with the wild-type strain (Tf-43037), while the sham-infected group exhibited minimal (baseline) bone loss (Fig. 8a and b). These data suggest that *T. forsythia*-secreted MGO, by dysregulating the activities of monocytes and fibroblasts, might be responsible for promoting alveolar bone loss. However, the effect of *T. forsythia*-derived MGO and glycated adducts on bone remodeling activities warrants further investigation.

## DISCUSSION

*T. forsythia* among the subgingival microbial community uniquely produces MGO. This electrophilic and highly reactive dicarbonyl compound can covalently modify amino acid side chains in proteins to generate glycated inflammatory adducts known as AGEs (4). Previous studies have shown that levels of MGO in gingival crevicular fluid from periodontally diseased sites correlate with the severity of pocket depth and levels of a red-complex consortium comprising *T. forsythia*, *P. gingivalis*, and *T. denticola* (4, 37). Given that *T. forsythia* is likely the sole bacterial source of MGO within the severely diseased pockets, we argued that *T. forsythia*-derived MGO is likely responsible for driving the disease severity by forming glycation adducts in host proteins. A previous study from our group showed that *T. forsythia* culture supernatants can modify collagen to glycated adducts (AGE collagen), which subsequently cause RAGE receptor-mediated secretion of inflammatory cytokines in monocytes (5). The current study addressed how *T. forsythia*-secreted MGO might impact periodontal tissue health. We investigated the effects of the wild-type *T. forsythia* and its isogenic mutant lacking the ability to secrete MGO on the functional roles of HGFs, endothelial cells, and neutrophils. As predicted, the wild-type strain secreting MGO formed AGE adducts with proteins present in the culture medium, and the levels of adducts were significantly higher than the culture supernatant of the MGO mutant. The wild-type culture supernatant-treated collagen, compared to the MGO mutant culture supernatant-treated collagen, induced increased apoptosis in HGFs and inhibited the migration of HGFs. Moreover, the culture supernatant from the wild-type strain, compared to that from the MGO-deficient strain, induced higher adhesion molecule expression in endothelial cells, resulting in increased adhesion of monocytes and endothelial transmigration of neutrophils. In a mouse model of periodontitis, *T. forsythia* infection resulted in an increased formation of AGE adducts in the gingival tissue and elevated alveolar bone loss in a manner dependent on the production of MGO. Taken together, the buildup of AGE adducts in the gingival tissue due to *T. forsythia* infection likely contributes to inflammation and alveolar bone loss.

In the current study, we found that Annexin V and PI-positive cell populations were higher when the human gingival fibroblasts were exposed to cell-free medium from the wild-type *T. forsythia* (Tf-43037). In addition, human gingival fibroblasts secreted pro-inflammatory cytokines TNF-α and IL-6 more robustly when challenged with the Tf-43037-modified collagen than the MGO mutant. MGO-modified AGE adducts via engagement of RAGE are known to induce ROS production to enhance cellular apoptosis (13). In the current study, we demonstrated that the wild-type Tf-43037 supernatant-treated collagen induced higher levels of ROS in HGFs than the MGO-deficient TFM-1726 supernatant-treated collagen.

GFs maintain periodontal tissue structural and functional integrity (38). In addition to this pivotal role in tissue remodeling, HGFs act as sentinel cells that modulate the gingival immune response to oral pathogen components by producing cytokines and other inflammatory mediators (39, 40). In this study, we demonstrated that human gingival fibroblasts respond to glycated collagen that might build up in gingival tissues due to *T. forsythia* infection by producing inflammatory cytokines TNF-α and IL-6. Furthermore, by expressing surface adhesion molecules, gingival fibroblasts can capture and accumulate monocytes at infection sites, thereby exacerbating inflammation. Increased apoptosis and inhibition of the migration ability of fibroblasts due to glycated adducts produced by *T. forsythia* MGO could further disrupt the routine repair and homeostasis of gingival tissue. Additionally, RAGE-AGE interaction has been shown to elevate the expression of adhesive molecules and promote chemotaxis and transendothelial migration of neutrophils to inflamed sites (41). Our data showed that AGE adducts formed by *T. forsythia* could increase the expression of endothelial cell adhesion molecules and the transmigration of neutrophils. High accumulation of AGEs in gingival tissues can contribute to chronic inflammation in periodontitis by enhancing the persistent recruitment of neutrophils to gingival tissues.

In summary, the contribution of *T. forsythia*-secreted MGO to the pathogenesis of periodontitis is evident in the oral gavage mouse model of periodontitis. Our data showed an increased accumulation of AGE adducts in mouse gingival tissues and alveolar bone loss after infection with the wild-type *T. forsythia* compared to the MGO-deficient mutant. This proof-of-principle study supports the contribution of AGE adducts that might build up in the gingival tissue as a result of chronic *T. forsythia* infection in the pathogenesis of periodontitis.

## SUPPLEMENTAL MATERIAL

Fig. S1a iai.00560-24-s0001.tif MGO deficiency impairs AGE formation ability in *T. forsythia*.

Fig. S1b iai.00560-24-s0002.tif SDS-PAGE profiles of the culture supernatants of the wild-type and mutant strains.

Fig. S2a iai.00560-24-s0003.tif *T. forsythia* secreted MGO upregulates adhesion molecules in HAoECs.

Fig. S2b iai.00560-24-s0004.tif *T. forsythia* secreted MGO upregulates adhesion molecules in HAoECs.

Fig. S3 iai.00560-24-s0005.tif Percentage of PI and Annexin-V positive cell population.

Supplemental material iai.00560-24-s0006.docx Legends for Fig. S1 to S3.

ASM does not own the copyrights to Supplemental Material that may be linked to, or accessed through, an article. The authors have granted ASM a non-exclusive, world-wide license to publish the Supplemental Material files. Please contact the corresponding author directly for reuse.
