# Supplementary figures and images for: Oral bacterium contributes to periodontal inflammation by forming advanced glycation end products
Source: Infect Immun. 2025 Apr 2;93(5):e00560-24. doi: 10.1128/iai.00560-24 (PMC12070732; doi:10.1128/iai.00560-24)

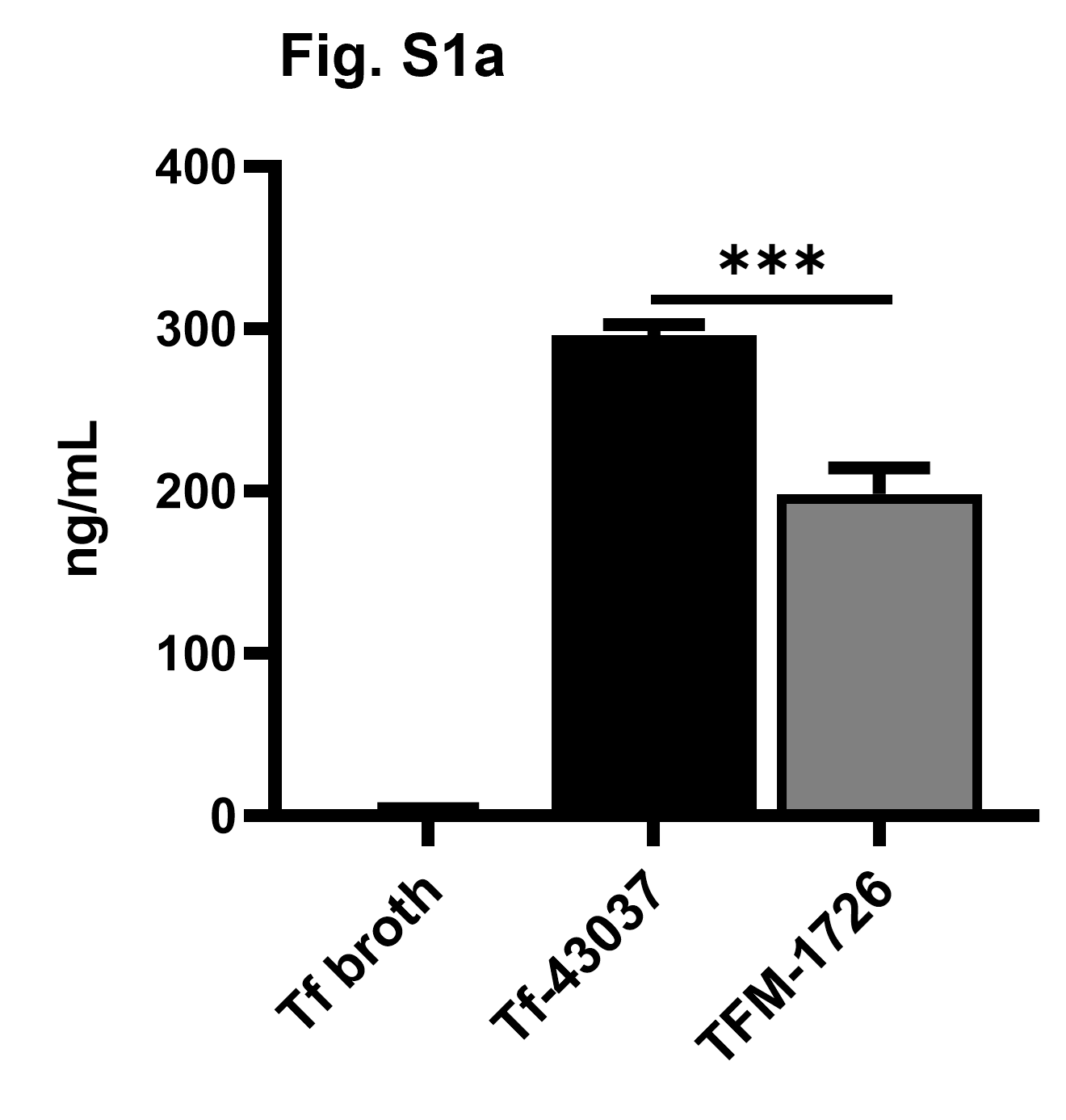

Supplement: Fig. S1a — MGO deficiency impairs AGE formation ability in T. forsythia. [file iai.00560-24-s0001.tif]

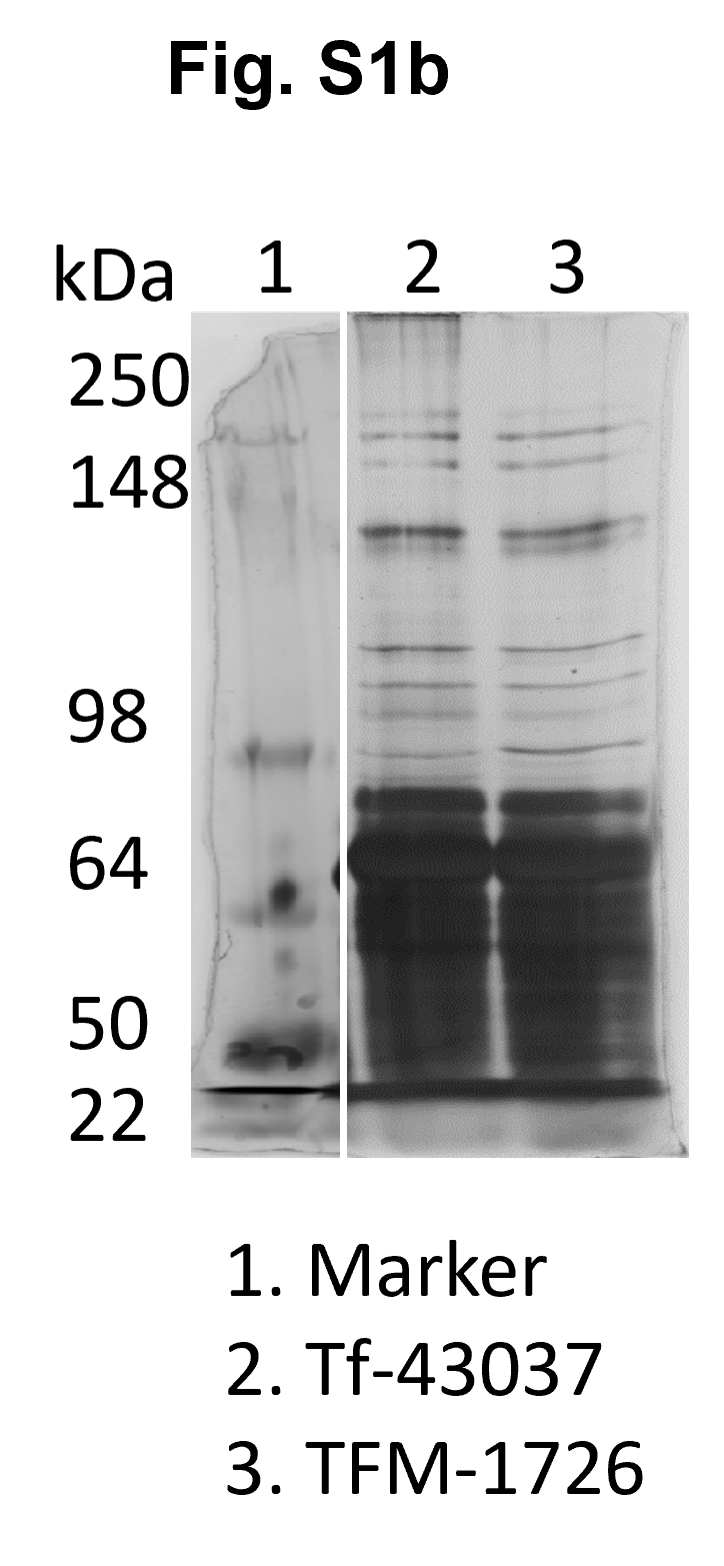

Supplement: Fig. S1b — SDS-PAGE profiles of the culture supernatants of the wild-type and mutant strains. [file iai.00560-24-s0002.tif]

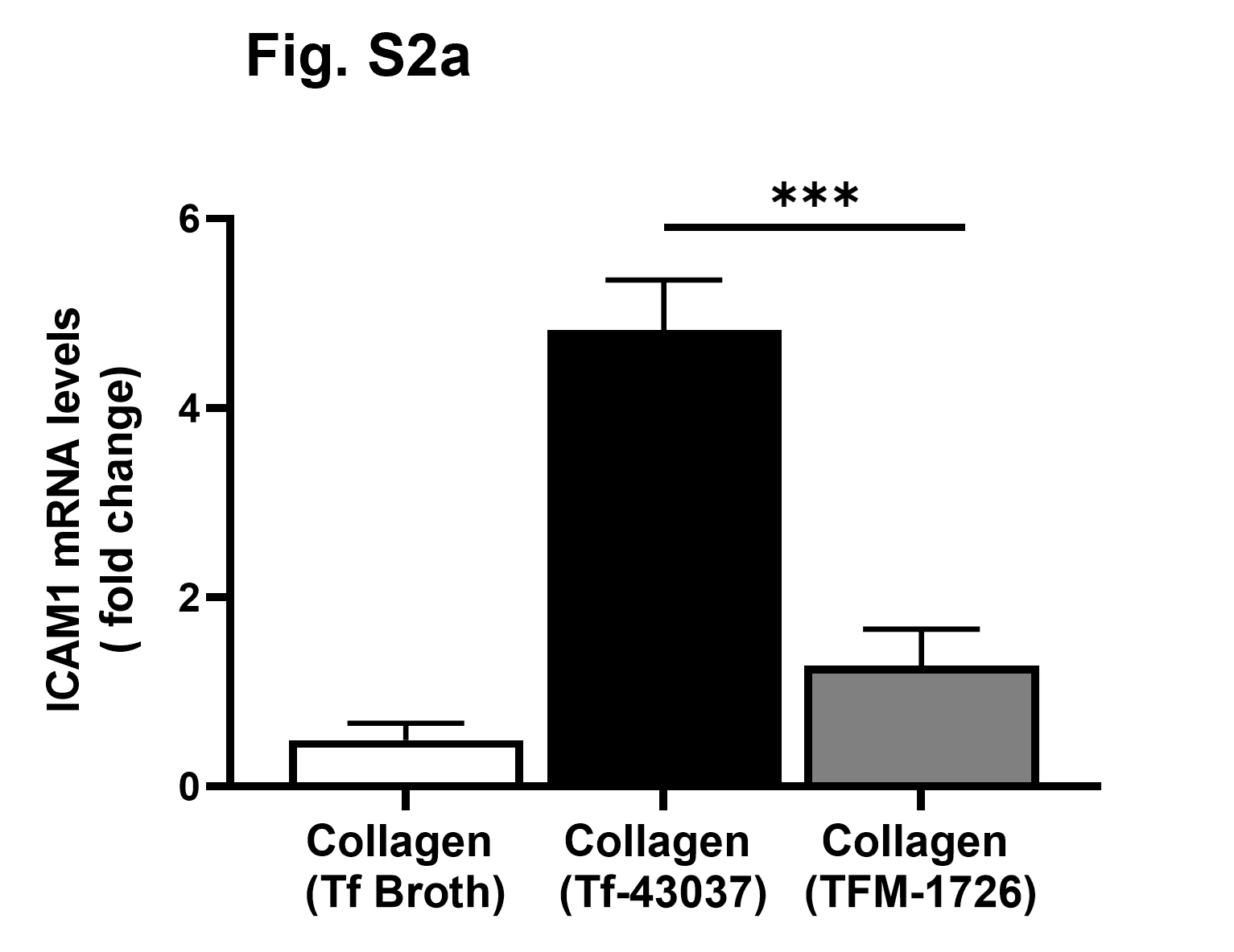

Supplement: Fig. S2a — T. forsythia secreted MGO upregulates adhesion molecules in HAoECs. [file iai.00560-24-s0003.tif]

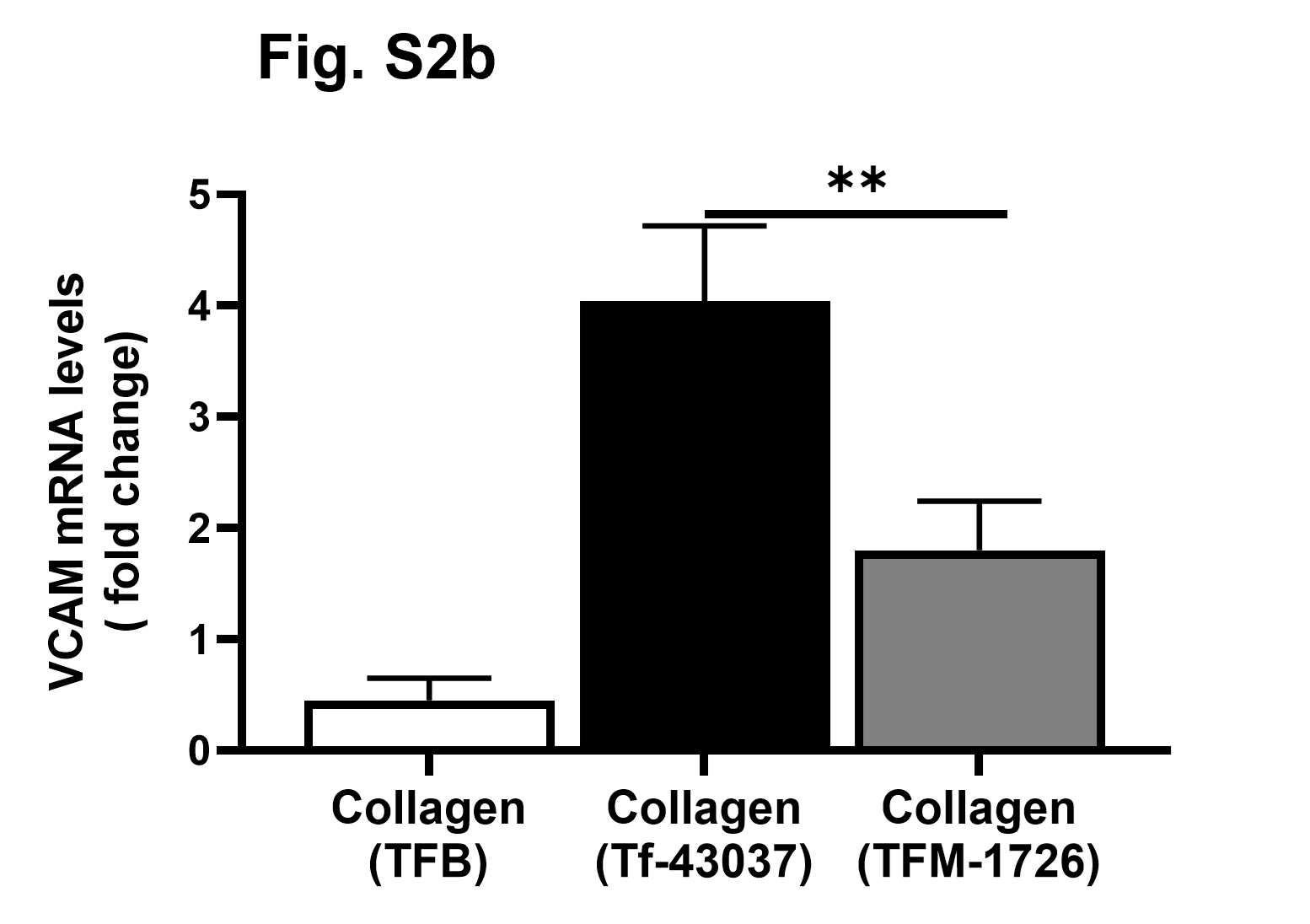

Supplement: Fig. S2b — T. forsythia secreted MGO upregulates adhesion molecules in HAoECs. [file iai.00560-24-s0004.tif]

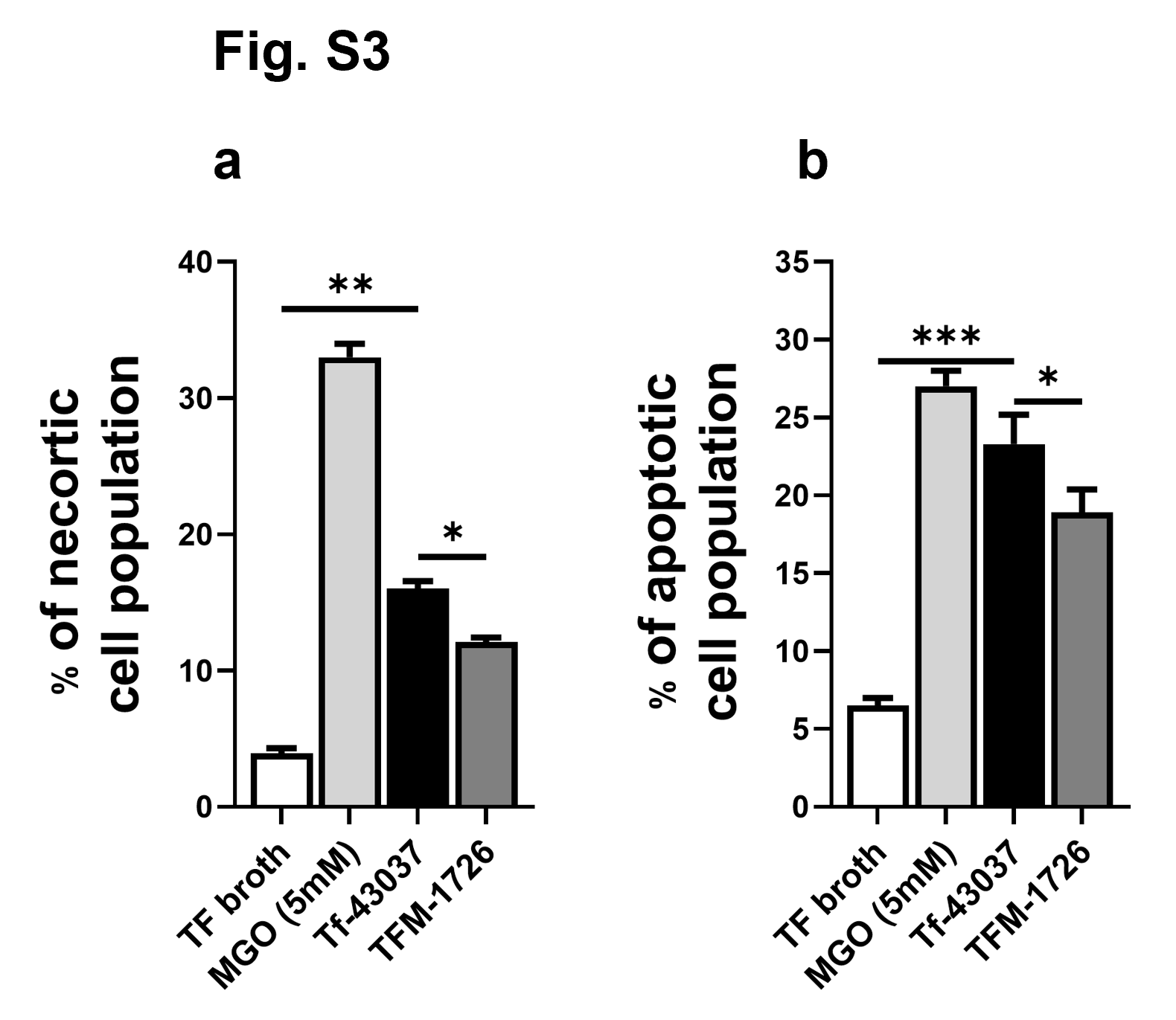

Supplement: Fig. S3 — Percentage of PI and Annexin-V positive cell population. [file iai.00560-24-s0005.tif]
