# Supplementary material for: Oral bacterium contributes to periodontal inflammation by forming advanced glycation end products
Source: Infect Immun. 2025 Apr 2;93(5):e00560-24. doi: 10.1128/iai.00560-24 (PMC12070732; doi:10.1128/iai.00560-24)
Supplement: Supplemental material — Legends for Fig. S1 to S3. [file iai.00560-24-s0006.docx]

**Fig. S1. MGO deficiency impairs AGE formation ability in *T. forsythia****.*

Cell-free culture supernatants were collected from both the wild-type (Tf-43037) and MGO mutant strain (TFM-1726). a) AGE levels in the supernatants were quantified with OxiSelect Advanced Glycation End Product Competitive ELISA Kit (catalog number STA-817, Cell Biolabs, Inc., San Diego, CA, USA) as per the manufacturer's instructions. The concentrations of AGEs were calculated using the standard curve of AGE-bovine serum albumin. Bars indicate mean ± SD. Statistically significant differences are indicated as *** *p* < 0.001. b). In addition, we performed SDS-PAGE silver staining for cell culture supernatants from both wild-type and MGO mutant strains and the data suggest that both the wild-type and mutant showed identical band profiles and relative amounts after silver staining.

**Fig. S2. *T. forsythia* secreted MGO upregulates adhesion molecules in HAoECs.**

Human aortic endothelial cells (HAoECs) were incubated with cell-free bacterial culture supernatants from Tf-43037 and TFM-1726 for 3 hrs. Total RNA was extracted from HAoECs and a) ICAM, b) VCAM adhesion molecules expression levels were quantified with specific primers. GAPDH was used as internal control. Gene expression was quantified as fold change relative to HGFs with medium alone and indicated mean± SD. Statistically significant differences indicated as ***, P<0.001, ** P<0.01.

**Fig.S3.** ***T. forsythia* secreted MGO promotes apoptosis and cell necrosis in gingival fibroblasts (HGFs).**

HGFs were seeded into a 12-well cell culture plate (0.5× 10^6^ cells/well) and incubated overnight. Cells were treated for 2 hrs. with 25 μl and 50 µL of cell-free culture supernatants from Tf-43037 or TFM-1726, TFB (20%v/v) and MGO (5mM). After washing the wells with cold PBS, cells were detached with trypsin-EDTA solution, collected by centrifugation and stained with FITC labeled Annexin-V and propidium iodide (PI). Results are representative from three independent experiments. Bar graphs (a) shows the percentage of positive cell population for PI (total of single and dual positive cells), (b) shows the percentage of positive cell population for Aneexin-V (total of single and dual positive cells). Bars represent mean ± s.d. ***P<0.001 **P<0.01, * P<0.05.
